# SYPL1 Inhibits Apoptosis in Pancreatic Ductal Adenocarcinoma *via* Suppression of ROS-Induced ERK Activation

**DOI:** 10.3389/fonc.2020.01482

**Published:** 2020-09-15

**Authors:** Yunda Song, Xuesong Sun, Fangting Duan, Chaobin He, Jiali Wu, Xin Huang, Kaili Xing, Shuxin Sun, Ruiqi Wang, Fengxiao Xie, Yize Mao, Jun Wang, Shengping Li

**Affiliations:** ^1^State Key Laboratory of Oncology in South China, Collaborative Innovation Center for Cancer Medicine, Sun Yat-sen University Cancer Center, Guangzhou, China; ^2^Department of Pancreatobiliary Surgery, Sun Yat-sen University Cancer Center, Guangzhou, China; ^3^Department of Nasopharyngeal Carcinoma, Sun Yat-sen University Cancer Center, Guangzhou, China

**Keywords:** apoptosis, ERK, pancreatic ductal adenocarcinoma, ROS, SYPL1

## Abstract

Synaptophysin-like 1 (SYPL1) is a neuroendocrine-related protein. The role of SYPL1 in pancreatic ductal adenocarcinoma (PDAC) and the underlying molecular mechanism remain unclarified. Here, after analyzing five datasets (GSE15471, GSE16515, GSE28735, TCGA, and PACA-AU) and 78 PDAC patients from Sun Yat-sen University Cancer Center, we demonstrated that SYPL1 was upregulated in PDAC and that a high level of SYPL1 indicated poor prognosis. Bioinformatics analysis implied that SYPL1 was related to cell proliferation and cell death. To validate these findings, gain-of-function and loss-of-function experiments were carried out, and we found that SYPL1 promoted cell proliferation *in vitro* and *in vivo* and that it protected cells from apoptosis. Mechanistic studies revealed that sustained extracellular-regulated protein kinase (ERK) activation was responsible for the cell death resulting from knockdown of SYPL1. In addition, bioinformatics analysis showed that the expression of SYPL1 positively correlated with antioxidant activity. Reactive oxygen species (ROS) were upregulated in cells with SYPL1 knockdown and vice versa. Upregulated ROS led to ERK activation and cell death. These results suggest that SYPL1 plays a vital role in PDAC and promotes cancer cell survival by suppressing ROS-induced ERK activation.

## Introduction

Pancreatic ductal adenocarcinoma (PDAC) is a lethal disease with a dismal 5-years survival rate of <10% in the USA (1) and is predicted to be the second leading cause of cancer-specific death by 2030 (2). Surgical resection can be performed in only ~20% of PDAC patients, whereas most patients suffer from locally advanced or metastatic diseases due to the insidious onset and early metastasis of PDAC. Even after resection with curative intent, nearly 80% of patients experience recurrence (3). To improve clinical outcomes, it is vital to deepen the understanding of PDAC mechanisms and identify new therapeutic targets.

Synaptophysin-like 1 (SYPL1), which belongs to the synaptophysin (SYP) family, was originally regarded as a neuroendocrine-related protein (4) and is expressed in both neuronal and non-neuronal tissues (5). SYPL1 is reported as a component of transport vesicles, which associate with insulin-responsive glucose transporter type 4 (GLUT4)-containing vesicles in adipocytes (6) and podocyte exosome-enriched fraction in urine (7). In addition, SYPL1 may be a regulator of the NF-κB pathway in a genome-wide siRNA screen (8). Recently, an immunohistochemistry (IHC)-based study showed that SYPL1 was a prognostic factor of poor prognosis of hepatocellular carcinoma and was related to epithelial-mesenchymal transition (9). In a bioinformatics-based study, SYPL1 was predicted to play a vital role in papillary thyroid carcinoma (10). However, the mechanism by which SYPL1 promotes the initiation and progression of tumors and the role of SYPL1 in PDAC remain unclarified.

In this study, significant upregulation of SYPL1 was detected in PDAC patient tumor tissue, which indicated poor prognosis. Knockdown of SYPL1 inhibited proliferation, induced apoptosis of tumor cells, and vice versa. Mechanistically, knockdown of SYPL1 increased reactive oxygen species (ROS) and sustainably activated extracellular-regulated protein kinases (ERKs). We demonstrated that SYPL1 promoted PDAC progression through its regulation of the ROS/ERK pathway.

## Materials and Methods

### Patients and Tissue Specimens

This study was approved by the ethics committee of Sun Yat-sen University Cancer Center (SYSUCC). Tissue samples were collected from 78 PDAC patients who underwent curative resections in SYSUCC from March 2008 to November 2017. Preoperative chemotherapy or radiotherapy was not performed. The last follow-up data were from October 14, 2019. Overall survival (OS) was defined as the duration from surgery to death or last follow-up. The clinical features of the patients are summarized in Table 1. After excision, tissue samples were formalin fixed and paraffin embedded.

**Table 1:** Characteristics of pancreatic ductal adenocarcinoma patients from Sun Yat-sen University Cancer Center.

**Clinicopathological features**	***n***	**SYPL1 expression**		***p*-values**
		**Low (*n* = 30)**	**High (*n* = 48)**	
**Sex**				0.744
Male	45	18	27	
Female	33	12	21	
**Age**		58.000 ± 9.229	59.042 ± 11.123	0.669
**Tumor site**				0.532
Head of pancreas	65	24	41	
Body and tail	13	6	7	
**Grade**				0.607
Moderate to good	35	12	23	
Poor to poor-moderate	39	17	22	
Not determine	4	1	3	
**Tumor diameter (cm)**		4.0 (3.0–5.0)	3.0 (3.0–4.5)	0.31
**Tumor extends beyond pancreas**				0.767
Presence	62	25	37	
Absence	12	4	8	
Not determine	4	1	3	
**Nodal status**				0.590
Positive	36	15	21	
Negative	42	15	27	
**Positive lymph node**		0.5 (0.0–2.0)	0 (0.0–1.0)	0.398
**Perineural invasion**				0.045
Presence	59	19	40	
Absence	19	11	8	
**Microvascular invasion**				0.713
Presence	21	9	12	
Absence	44	21	23	

### Immunohistochemical Staining and Scoring

IHC staining was carried out as we described before (11). In short, after antigen retrieval by microwave treatment in citrate buffer (pH 6.0), paraffin-embedded 4 mm tissue sections were incubated with a rabbit anti-SYPL1 monoclonal antibody (ab184176, 1:150 dilution; Abcam, Cambridge, MA, USA) for 2 h at room temperature and stained with 3,3′-diaminobenzidine (DAB) after incubation with secondary antibody. All specimens were evaluated using Image-Pro Plus 6.0 software. The mean integrated optical density (mean IOD) was calculated according the following formula: mean IOD = IOD/area (12, 13).

### Public Databases

PDAC expression data and clinical information were acquired from Gene Expression Omnibus (GEO, datasets GSE15471, GSE16515, and GSE28735, https://www.ncbi.nlm.nih.gov/geo), the Cancer Genome Atlas (TCGA, https://portal.gdc.cancer.gov) (14), the International Cancer Genome Consortium (ICGC, dataset PACA-AU, only array-based gene data were included, https://dcc.icgc.org) and the Genotype-Tissue Expression (GTEx, https://www.gtexportal.org/home). Gene Expression Profiling Interactive Analysis (GEPIA, http://gepia.cancer-pku.cn/) was also used. For pathway analysis, gene sets for Kyoto Encyclopedia of Genes and Genomes (KEGG) pathway and Gene Ontology (GO) analyses were downloaded from https://www.gsea-msigdb.org/gsea/index.jsp.

### Cell Culture and Transfection

BxPC-3, CFPAC-1, and PANC-1 human PDAC cell lines were purchased from the Cell Bank of the Chinese Academy of Sciences (Shanghai, China), and the immortal human pancreatic duct epithelial cell line HPDE6-C7 was provided as a gift from Professor Dongxin Lin from SYSUCC. Cells were cultured at 37°C in a humidified atmosphere of 5% CO_2_. The culture medium was used as recommended and was supplemented with 10% fetal bovine serum (Gibco, California, USA).

Cell lines with SYPL1 stably silenced or overexpressed were established using lentiviruses (iGeneBio, GuangZhou, China). We also used siRNA (RiboBio, Guangzhou, China), which was transfected into cells by Lipofectamine 2000 (Invitrogen, California, USA) according to the manufacturer's instructions. To knockdown SYPL1, the following validated target sequence was used: CCTCATAGGCGATTACTCT.

### Reverse Transcription-Quantitative Polymerase Chain Reaction (RT-qPCR)

To extract total RNA, TRIzol reagent (15596026; Invitrogen, Carlsbad, CA, USA) was used. Reverse transcription was performed using qPCR RT Master Mix (FSQ-301; TOYOBO Co., Osaka, Japan). To determine the expression of RNA, SYBR® Green PCR Master Mix (QPS-201; Toyobo, Osaka, Japan) was used. The primer pairs were as follows:

SYPL1: CTTTGGCTCTGTGACCAGTATGG (forward), GATGGACTGTGTAGGCTGGTCT (reverse);
β-actin: CACCATTGGCAATGAGCGGTTC (forward), AGGTCTTTGCGGATGTCCACGT (reverse).

### Western Blot (WB)

WB was carried out as we described previously (11, 15). The antibodies used for WB included anti-SYPL1 monoclonal antibody (ab184176, 1:1500 dilution; Abcam, Cambridge, MA, USA), phospho-MAPK Family Antibody Kit (9910; Cell Signaling Technology [CST], Danvers, MA, USA), p44/42 MAPK (Erk1/2) (137F5) Rabbit mAb (4695; CST) and alpha tubulin antibody (11224-1-AP, Proteintech). Proteins were visualized using an enhanced chemiluminescence kit (4AW011; purchased from 4A Biotech Co., Ltd, Beijing, China).

### Cell Proliferation and Colony Formation Assay

Cell proliferation was assessed using the Cell Counting Kit-8 assay (CCK8, Dojindo, Tabaru, Japan) following the instructions. Cells were seeded in 96-well plates (1,000–2,000 cells/well). The incubation time of the CCK8 solution was 2 h.

Assessments of colony formation were performed as described before (11). Cells were seeded in 6-well plates (500 cells/well) and cultured for 2 weeks.

### Apoptosis and ROS Assay

Cells were plated in 6-well plates at 5 × 10^5^ cells/well and grew to confluence. Then cells were incubated with cisplatin (KGR0036, KeyGen Biotech, Nanjing, China) for 2 h. Cells were trypsinized and washed twice with phosphate-buffered saline (PBS). We used the Annexin V-APC/PI Apoptosis Detection Kit (KGA1030-100, KeyGen Biotech, Nanjing, China) to assess apoptosis according to the manufacturer's instructions. Data were acquired using flow cytometry.

For the ROS assay, the ROS fluorescence probe-DHE (KGAF019) was obtained from KeyGen BioTech (Nanjing, China) and flow cytometry was applied.

### Animal Experiments

All animal experiments were approved by the ethics committee of SYSUCC. Six-weeks-old Crj:BALB/c female athymic nude mice were purchased from Vital River (Beijing, China). A xenograft model was established by subcutaneously injecting 5 × 10^6^ cells into the left flank of the nude mice. We measured the size of the tumor every 7 days using calipers (volume = 0.5 × length × width × width). After 5–7 weeks, mice were euthanized, and xenografts were removed, weighed and preserved.

### Statistical and Bioinformatics Analysis

Gene set enrichment analysis (GSEA) was performed using software downloaded from https://www.gsea-msigdb.org/gsea/index.jsp (16, 17). Gene set variation analysis (GSVA) was carried out using the R package “GSVA” (18). GSEA and GSVA were used in pathway analysis. Details of the statistical analysis were described in our previous study (19). Graph Pad Prism 7.0 software, R 3.5.1 and SPSS 23 were used.

## Results

### SYPL1 Is Upregulated in PDAC

**Figure 1:**
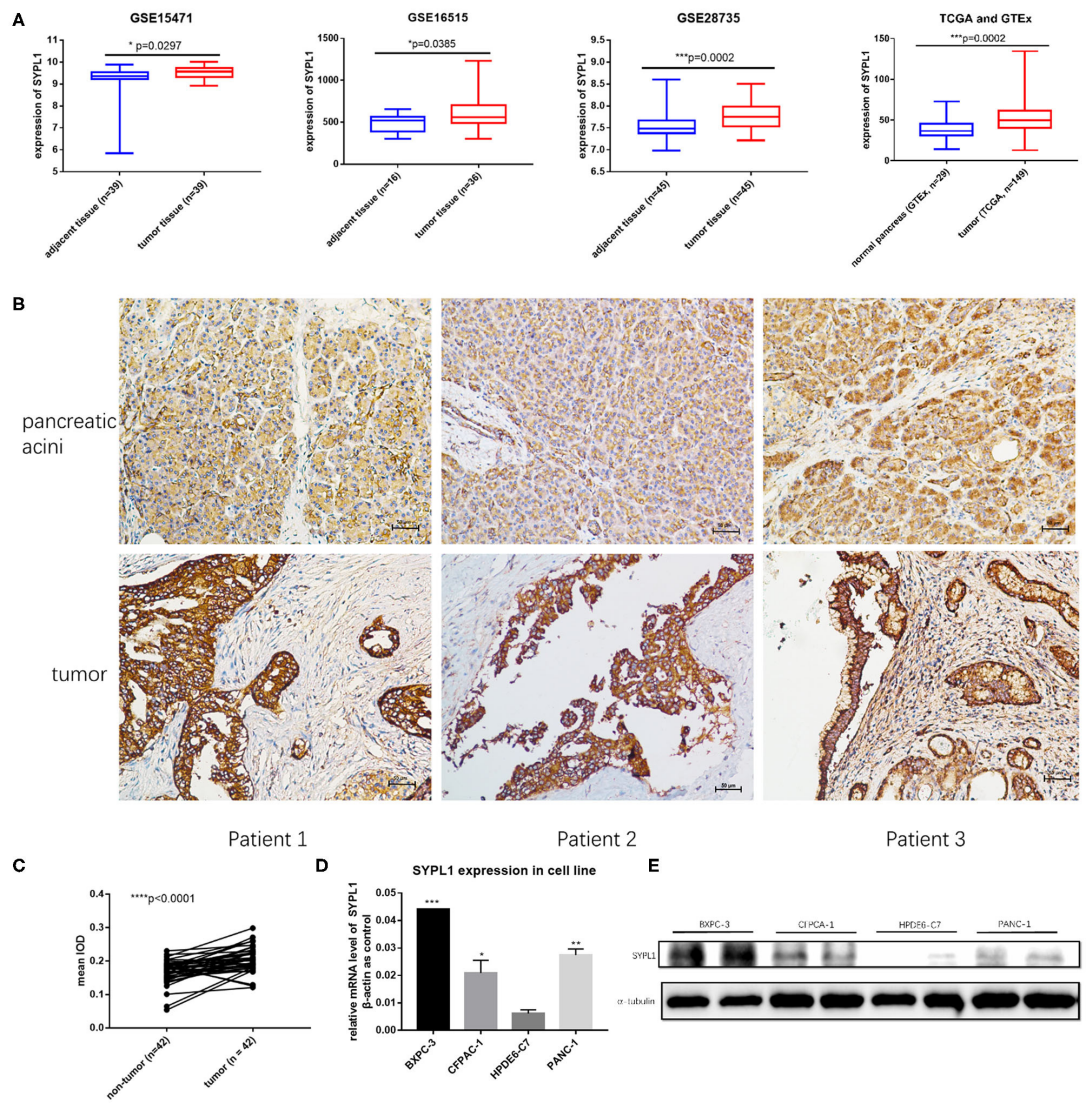
SYPL1 was upregulated in pancreatic ductal adenocarcinoma (PDAC). **(A)** SYPL1 was upregulated in PDAC at the transcription level (GSE15471, GSE16515, GSE28735, TCGA, and GTEx datasets were used). **(B)** The expression of SYPL1 in tumor and pancreatic acini at the protein level. Tissue sections from three PDAC patients were used as examples. **(C)** Quantification of SYPL1 protein levels in 42 paired tumor sections and pancreatic acini sections. **(D)** Quantification of SYPL1 RNA in PDAC cell lines (BXPC-3, CFPAC-1, and PANC-1) and immortal human pancreatic duct epithelial cell lines HPDE6-C7. **(E)** SYPL1 protein levels in cell lines. *p < 0.05, ***p < 0.001 and ****p < 0.0001.

We analyzed the gene expression of SYPL1 in GEO (GSE15471, GSE16515, and GSE28735), TCGA and GTEx. SYPL1 was elevated in tumor tissue at the level of transcription (Figure 1A). Forty-two tissue sections from SYSUCC contained both PDAC and pancreatic acini. IHC results confirmed the upregulation of SYPL1 in PDAC at the protein level (Figures 1B,C). SYPL1 was also elevated in PDAC cell lines compared to its expression in the HPDE6-C7 pancreatic duct epithelial cell line based on WB and qPCR assays (Figures 1D,E).

### Elevated SYPL1 Indicates Poor Prognosis

**Figure 2:**
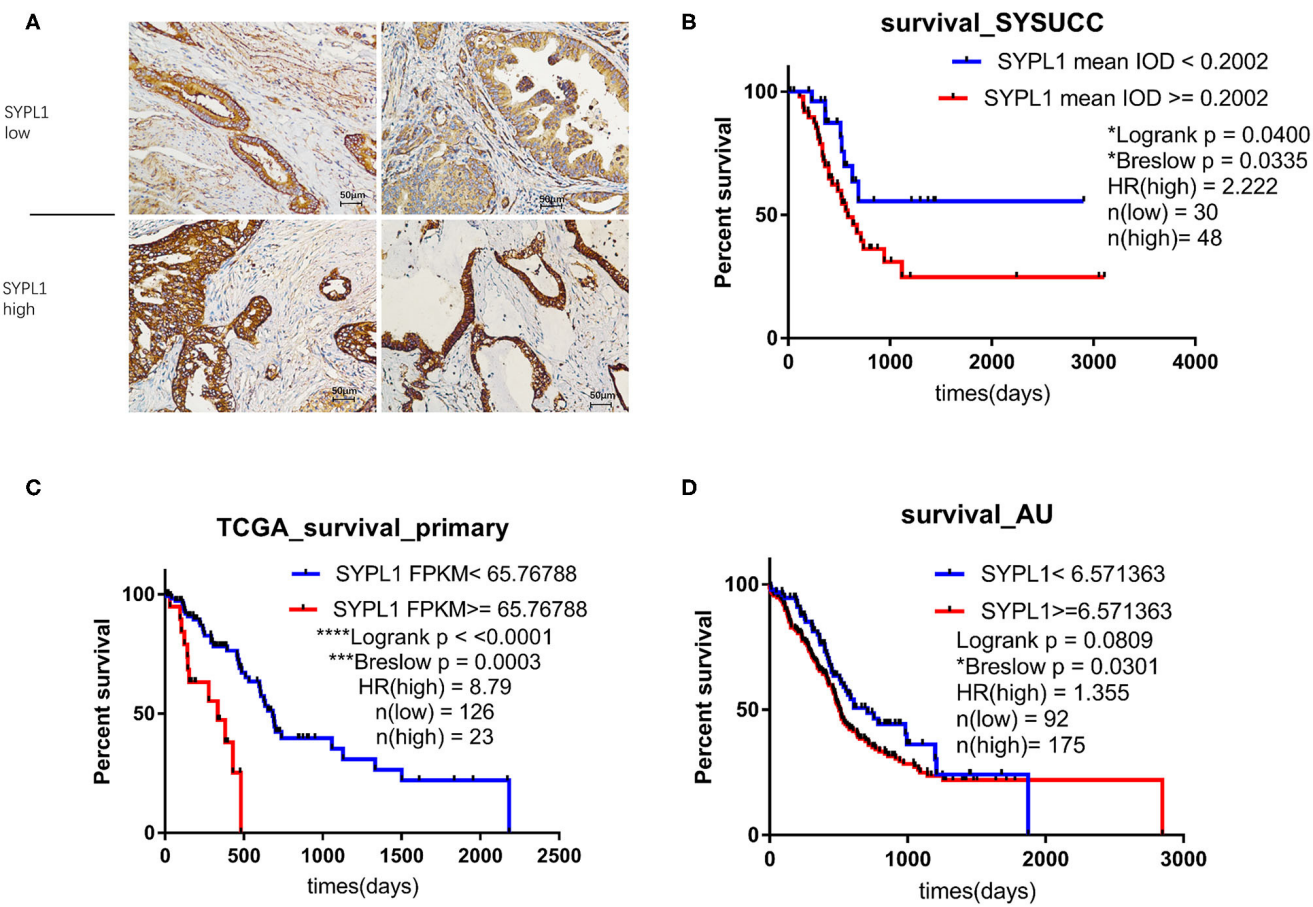
Upregulation of SYPL1 indicated poor prognosis. **(A)** Two examples of SYPL1-high and SYPL1-low tumor sections. **(B)** Prognostic effects of SYPL1 on patients from Sun Yat-sen University Cancer Center. **(C)** Prognostic effects of SYPL1 for patients from TCGA. **(D)** Prognostic effects of SYPL1 for patients from PACA-AU.

We classified 78 tumor tissues of SYSUCC into 2 groups: SYPL1 low (mean IOD < 0.2002) and SYPL1 high (mean IOD ≥ 0.2002) (Figure 2A). The correlation between SYPL1 expression and clinical features is shown in Table 1. SYPL1 positively correlated with perineural invasion (*p* = 0.045). Patients with upregulated SYPL1 had shorter OS (Figure 2B). Similar results were observed in TCGA and PACA-AU (Figures 2C,D, Supplementary Figure 2). Besides, in TCGA dataset, SYPL1 positively correlated with tumor size (Supplementary Figure 5). Univariate and multivariate analysis using the COX model showed that the grade of PDAC (hazard ratio [HR]: 2.924, 95% confidence interval [CI]: 1.291–6.621), tumor size (HR: 3.900, 95% CI: 1.114–13.645) and the expression level of SYPL1 (HR: 2.807, 95% CI: 1.204–6.543) were independent prognostic factors for patients in the SYSUCC (Table 2).

**Table 2:** Univariate and multivariate Cox regression analysis for patients from Sun Yat-sen University Cancer Center.

	**Univariate analysis**			**Multivariate analysis**		
	**HR**	**95% CI**	***p*-values**	**HR**	**95% CI**	***p*-values**
Sex	1.257	0.643–2.459	0.504			
Age	1.017	0.982–1.054	0.341			
Location	1.387	0.573–3.356	0.468			
Grade (poor to poor-moderate)	2.447	1.214–4.935	**0.012**	2.924	1.291–6.621	0.010
Diameter (>2.75 cm)	5.282	1.607–17.365	**0.006**	3.900	1.114–13.645	0.033
Tumor extends beyond pancreas	2.501	0.870–7.189	0.089			
Perineural invasion	1.285	0.562–2.940	0.552			
Microvascular invasion	2.084	0.943–4.607	0.070			
Positive nodal status	2.268	1.156–4.450	**0.017**	1.363	0.634–2.932	0.428
SYPL1	2.231	1.015–4.903	**0.046**	2.807	1.204–6.543	0.017

*The bold values indicates p < 0.05*.

### Explorations of KEGG Pathways Related to SYPL1

**Figure 3:**
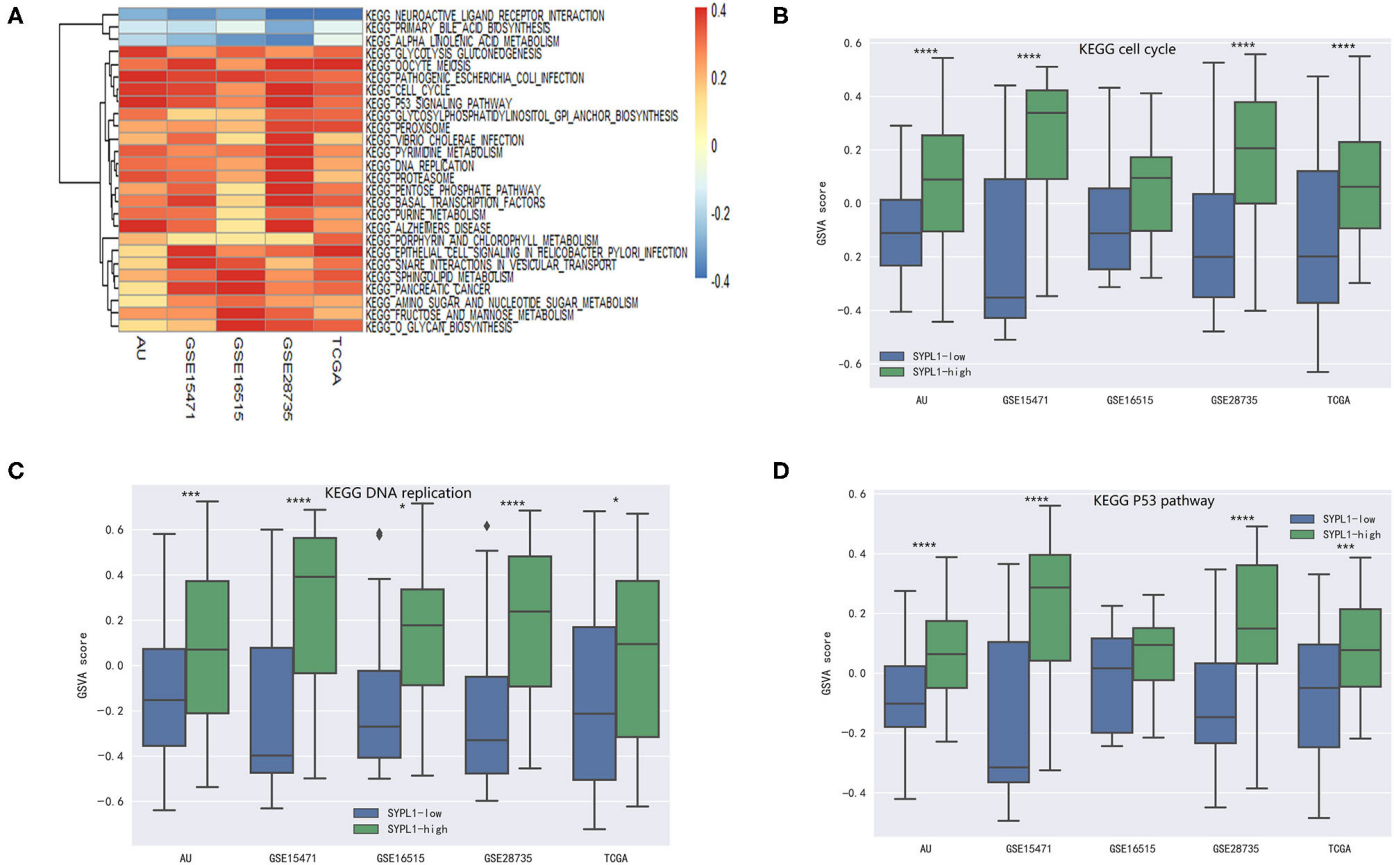
SYPL1 was related to cell proliferation and apoptosis. **(A)** Correlation coefficients (*r*) between SYPL1 and GSVA scores of KEGG pathways were calculated. Twenty-six pathways with *r* > 0.1 in GSE15471, GSE16515, GSE28735, TCGA, and PACA-AU or pathways with *r* < −0.1 in these datasets were identified as pathways related to SYPL1. **(B)** The GSVA score of the KEGG cell cycle gene set in SYPL1-high and SYPL1-low samples. **(C)** The GSVA score of the KEGG DNA replication gene set in SYPL1-high and SYPL1-low samples. **(D)** The GSVA score of the KEGG P53 pathway gene set in SYPL1-high and SYPL1-low samples (Both tumor tissues and adjacent tumor tissues of GSE15471, GSE16515, and GSE28735 were taken into analysis in **(B–D)**, while TCGA and PACA-AU included only tumor tissues). *p < 0.05, **p < 0.01, ***p < 0.001, and ****p < 0.0001.

Five datasets (GSE15471, GSE16515, GSE28735, TCGA, and PACA-AU) were analyzed. Pathway activity was calculated using GSVA. Correlation coefficients (*r*) between SYPL1 and the activity of KEGG pathways were calculated. Twenty-six pathways with *r* > 0.1 in 5 datasets or pathways with *r* < −0.1 in 5 datasets were identified as pathways related to SYPL1 (Figure 3A). Two groups was defined based on SYPL1 expression (upper third vs. bottom third) in each dataset. The activities of the cell cycle pathway, DNA replication pathway and p53 pathway were higher in the SYPL1-high group, which indicated that SYPL1 was related to the proliferation and survival of PDAC cells (Figures 3B–D, Supplementary Figures 1,2).

### Altered SYPL1 Expression Affects Cell Proliferation *in vitro* and *in vivo*

**Figure 4:**
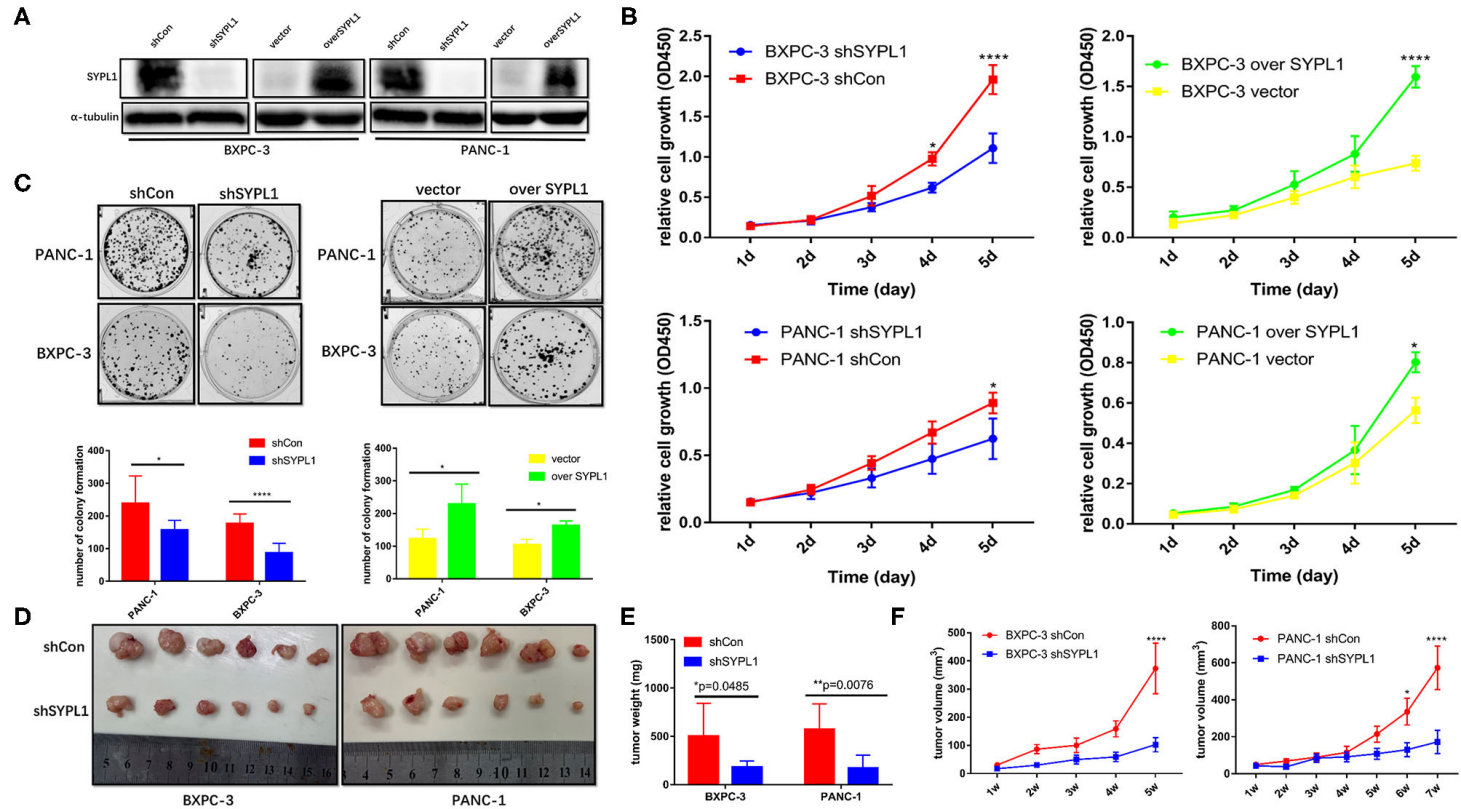
SYPL1 promoted cell proliferation. **(A)** Western blotting showed knockdown and overexpression of SYPL1 in BXPC-3 and PANC-1 cells. **(B)** CCK8 assay showed that knockdown of SYPL1 suppressed cell proliferation and vice versa. **(C)** Colony formation assay showed that knockdown of SYPL1 significantly reduced the colony formation ability and vice versa. **(D)** Suppression of SYPL1 significantly inhibited tumor growth as determined by a subcutaneous xenograft mouse model. **(E)** Tumor weight of the indicated cells after nude mice were sacrificed. **(F)** Curves of tumor diameter after the indicated cells were implanted in the subcutaneous xenograft mouse model. *p < 0.05, **p < 0.01 and ****p < 0.0001.

We infected BXPC-3 and PANC-1 cells with lentivirus carrying shRNA targeting SYPL1 (shSYPL1) or control shRNA (shCon). We infected the two cell lines with lentivirus carrying SYPL1 expression vector (overSYPL1) or control empty vector (Figure 4A). We also used siRNA to knockdown SYPL1 (Supplementary Figure 3). The CCK8 assay showed that knockdown of SYPL1 significantly reduced the proliferation of BXPC-3 and PANC-1 cells, while overexpression of SYPL1 did the opposite (Figure 4B). In the colony formation assay, we found that knockdown of SYPL1 significantly reduced the colony formation ability of BXPC-3 and PANC-1 cells and that overexpression of SYPL1 did the opposite (Figure 4C, Supplementary Figure 3). To assess the effects of SYPL1 on cell proliferation *in vivo*, a subcutaneous tumor model in BALB/c nude mice was established. Knockdown of SYPL1 in tumor cells slowed the growth of tumors (Figures 4D–F).

### Altered SYPL1 Expression Affects Cell Apoptosis

**Figure 5:**
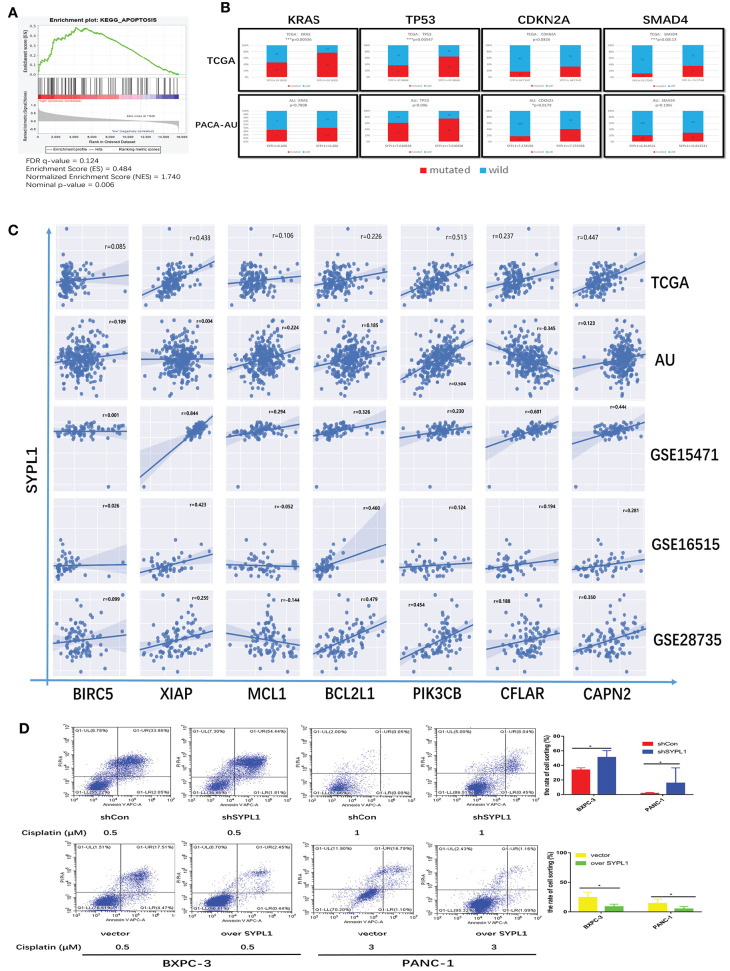
SYPL1 protected cells from apoptosis. **(A)** GSEA analysis showed that the expression of SYPL1 was related to the KEGG apoptosis pathway in the TCGA dataset. **(B)** In the TCGA and PACA-AU dataset, the mutation rates of KRAS, TP53, CDKN2A, and SMAD4 tended to be higher in SYPL1-high patients. **(C)** The expression of SYPL1 positively correlated with BIRC5, XIAP, MCL1, BCL2L1, PIK3CB, CFLAR, and CAPN2, which were reported as anti-apoptosis genes. **(D)** Flow cytometry showed that overexpression of SYPL1 protected cells from apoptosis, and vice versa. *p < 0.05 and **p < 0.01.

By performing GSEA of the TCGA dataset, we reconfirmed that the RNA level of SYPL1 was related to apoptosis (Figure 5A). High expression of SYPL1 indicated increased mutation frequencies of KRAS, TP53, CDKN2A, and SMAD4 in both TCGA and PACA-AU datasets (Figure 5B). These mutations were reported to protect tumor cells from apoptosis (20–25). We also analyzed the correlations between SYPL1 expression and some reported anti-apoptosis genes in five datasets (GSE15471, GSE16515, and GSE28735, TCGA, PACA-AU) (26–33). Generally, SYPL1 expression positively correlated with the expression of these anti-apoptosis genes (Figure 5C). To validate the anti-apoptotic role of SYPL1, cells were treated with cisplatin, an apoptosis inducer. Overexpression of SYPL1 reduced the percentage of apoptotic cells and vice versa (Figure 5D).

### Knockdown of SYPL1 Promoted Apoptosis by Activating ERK

**Figure 6:**
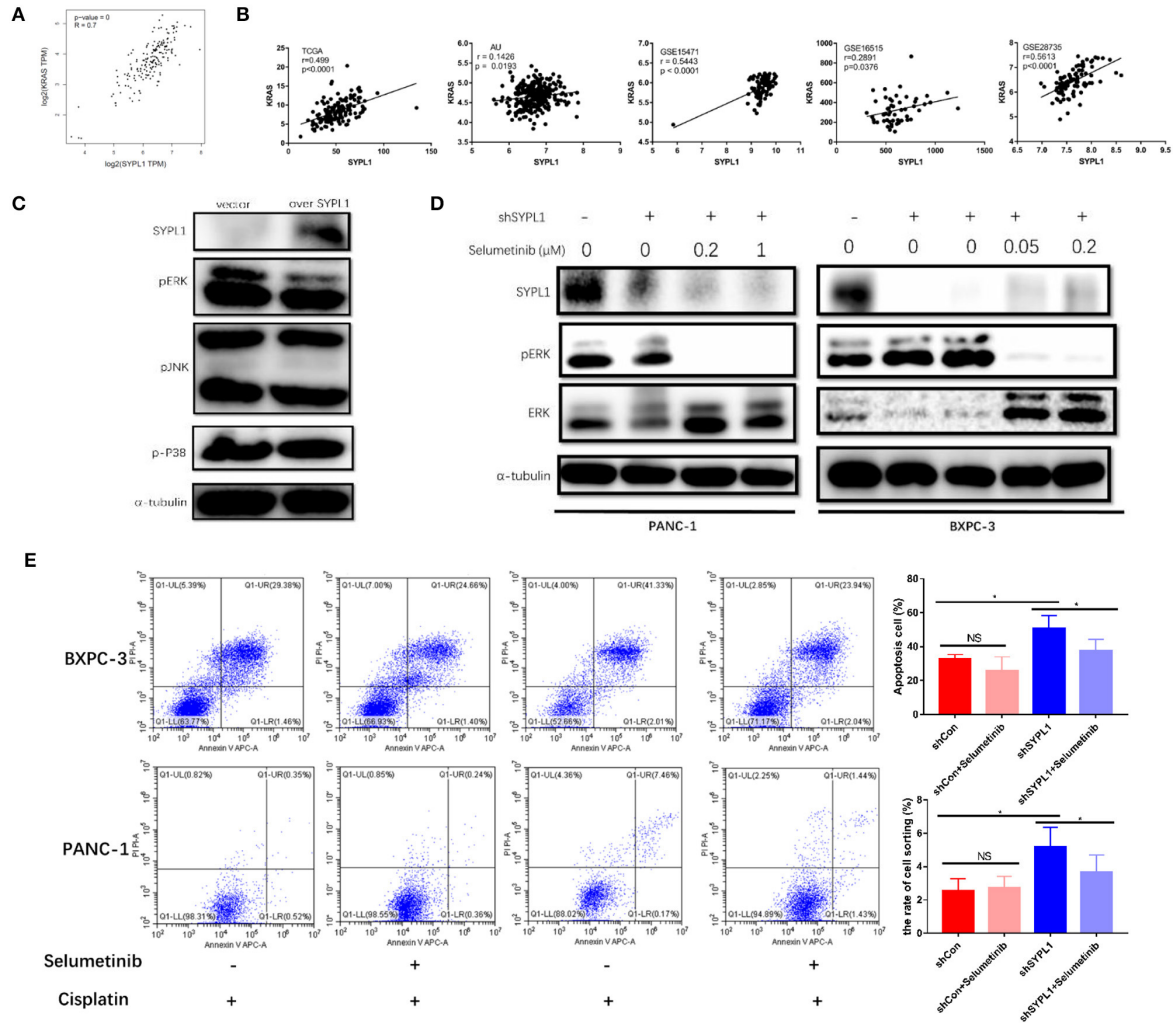
Knockdown of SYPL1 led to cell death by activating ERK. **(A)** We found that KRAS was one of the genes with the highest positive correlation to SYPL1 in the GEPIA database. **(B)** The positive correlation between the expression of SYPL1 and KRAS was validated in GSE15471, GSE16515, GSE28735, and PACA-AU. **(C)** When SYPL1 was overexpressed, phosphorylated ERK (pERK) was downregulated, while phosphorylated P38 and JNK did not change significantly. **(D)** In BXPC-3 and PANC-1 cells with knockdown of SYPL1, upregulation of pERK and downregulation of total ERK were observed, which indicated ERK activation. Selumetinib counteracted the activation of ERK. **(E)** Flow cytometry showed that selumetinib protected cells from downregulated SYPL1-induced cell death. *p < 0.05.

To explore the regulatory mechanisms of SYPL1, we searched for genes closely correlated with SYPL1 in pancreatic adenocarcinoma datasets of GEPIA. We found that the expression of KRAS was positively correlated with SYPL1 (*r* = 0.7), and similar results were seen in other datasets (Figures 6A,B), which implied that SYPL1 was related to the MAPK pathway. Phosphorylated ERK (pERK), phosphorylated P38 (p-P38) and phosphorylated JNK (pJNK) were assessed using WB. pERK was downregulated in BXPC-3 cells overexpressing SYPL1, while no obvious change was observed in pJNK or p-P38 (Figure 6C). In addition, upregulated pERK and downregulated ERK were observed in shSYPL1 cells, which indicated that ERK was sustainably activated in cells with SYPL1 knockdown (Figure 6D, Supplementary Figure 4). To clarify whether downregulation of SYPL1 inhibited proliferation and promoted apoptosis *via* sustainable ERK activation, selumetinib (AZD6244, Selleck Chemicals, Houston, TX, USA, 0.4 μM for BXPC-3, 2 μM for PANC-1), a MEK inhibitor, was used (Figure 6D). After shSYPL1 cells were treated with selumetinib, no obvious change was observed in cell proliferation (Supplementary Figure 3). However, selumetinib significantly reduced the number of apoptotic cells in the shSYPL1 cell line (Figure 6E), which meant that knockdown of SYPL1 resulted in apoptosis through sustainable ERK activation.

### Knockdown of SYPL1 Activates ERK by Increasing ROS

**Figure 7:**
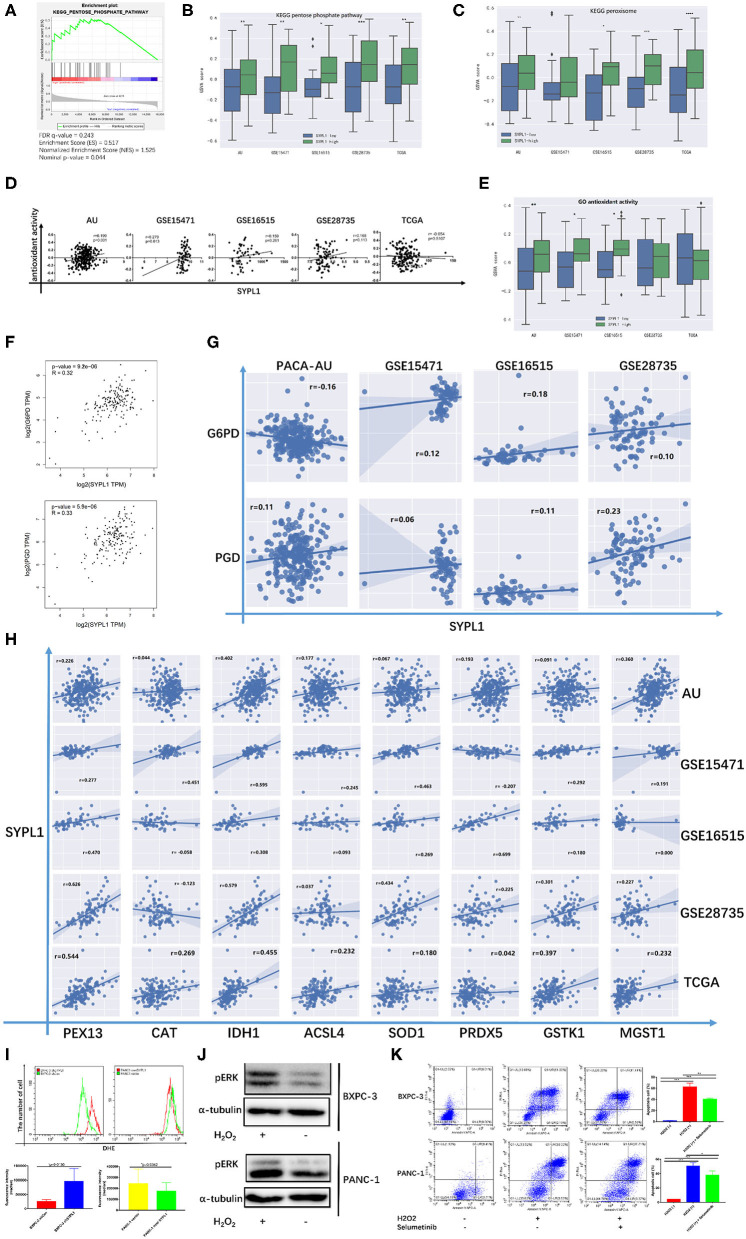
Knockdown of SYPL1 activated ERK through elevation in ROS. **(A)** GSEA analysis showed that the expression of SYPL1 was related to the KEGG pentose phosphate pathway (PPP) in the TCGA dataset. **(B)** The GSVA score of the PPP gene set in SYPL1-high and SYPL1-low samples. **(C)** The GSVA score of the peroxisome gene set in SYPL1-high and SYPL1-low samples (Both tumor tissues and adjacent tumor tissues of GSE15471, GSE16515, and GSE28735 were taken into analysis in **(B,C)**, while TCGA and PACA-AU included only tumor tissues). **(D)** The expression of SYPL1 was positively correlated with the GSVA scores of the GO antioxidant activity gene set. **(E)** The GSVA score of the antioxidant activity gene set in SYPL1-high and SYPL1-low patients (only tumor tissues were included). **(F)** In GEPIA, the expression of SYPL1 positively correlated with G6PD and PGD, enzymes generating NADPH in PPP. **(G)** The expression of SYPL1 positively correlated with G6PD and PGD in the other four datasets. **(H)** The expression of SYPL1 positively correlated with PEX13, CAT, IDH1, ACSL4, SOD1, PRDX5, GSTK1, and MGST1, which were reported to be antioxidants. **(I)** Flow cytometry showed that knockdown of SYPL1 led to upregulated ROS and vice versa. **(J)** Hydrogen peroxide upregulated phosphorylated ERK. **(K)** Hydrogen peroxide led to apoptosis of cells. *p < 0.05, **p < 0.01, ***p < 0.001 and ****p < 0.0001.

Several studies reported that sustainable activation of ERK, which promoted cell death, resulted from increased ROS (34–39). In Figure 3A, GSVA scores of both the pentose phosphate pathway (PPP) and peroxisome were positively correlated with the RNA level of SYPL1, which indicated that SYPL1 was involved in oxidative stress. GSEA on TCGA reconfirmed the relationship between SYPL1 and PPP, which was a major source of NADPH (Figure 7A). Increased GSVA scores of PPP were observed in SYPL1-high samples (Figure 7B). Glucose-6-phosphate dehydrogenase (G6PD) and phosphogluconate dehydrogenase (PGD) are NADPH-producing enzymes in the PPP (40). Generally, SYPL1 was positively correlated with both G6PD and PGD at the transcriptional level (Figures 7F,G). The GSVA score of peroxisomes was higher in SYPL1-high samples (Figure 7C). Peroxisomes have protective measures to counteract oxidative stress (41). At the transcriptional level, SYPL1 positively correlated with genes reported to possess antioxidant activity in the peroxisome gene set (Figure 7H) (41–46). In addition, a gene set named “GO_ANTIOXIDANT_ACTIVITY,” which contained components that can trap free radicals, was analyzed. The GSVA score of antioxidant activity was positively correlated with SYPL1, and a higher GSVA score was seen in SYPL1-high patients (Figures 7D,E). The results above suggested that SYPL1 was likely to help cope with oxidative stress. To validate this hypothesis, we used flow cytometry to assess intracellular ROS. The ROS level of SYPL1-silenced BXPC-3 cells was upregulated compared to that of shCon BXPC-3 cells, while PANC-1 cells overexpressing SYPL1 had decreased ROS levels (Figure 7I). Then, BXPC-3 and PANC-1 cells were treated with hydrogen peroxide (H_2_O_2_), which was reported to increase intracellular ROS and mimicked the effect of downregulated SYPL1 on intracellular ROS (47). Upregulation of pERK and increased apoptotic cells were found in H_2_O_2_-treated cells, and Selumetinib significantly reduced the number of apoptotic cells (Figures 7J,K, Supplementary Figure 4). Overall, knockdown of SYPL1 upregulated ROS, which led to activation of ERK and cell death.

## Discussion

The present study demonstrates that SYPL1, which is upregulated in tumor tissue and PDAC cell lines at the transcriptional level and protein level, is an independent factor associated with poor prognosis. Data analysis based on five datasets (GSE15471, GSE16515, GSE28735, TCGA, and PACA-AU) shows that SYPL1 is associated with the proliferation and survival of cancer cells. SYPL1 is silenced or upregulated in BXPC-3 and PANC-1 cells. Through CCK-8, colony formation and subcutaneous xenotransplanted tumor models in nude mice, we found that downregulation of SYPL1 inhibits tumor growth *in vitro* and *in vivo*, while overexpressed SYPL1 promotes cell proliferation. Data analysis and flow cytometry show that SYPL1 protects tumor cells from apoptosis. The prosurvival effects of SYPL1 result from the suppression of sustainably activated ERK by intracellular ROS. To our knowledge, this is the first study to demonstrate the enhancing effects of SYPL1 on cell proliferation and survival in tumors. This is also the first study that reports the expression of SYPL1 in PDAC and clarifies the mechanism of its anti-apoptotic effects.

The MAPK-ERK pathway, which requires delicate regulation of its spatiotemporal activity, is a double-edged sword in tumorigenesis. Activated ERK promotes cell survival as a result of its oncogenic potential. Paradoxically, aberrant activation of ERK is reported to promote cell death (48–50). The proapoptotic effect of the MAPK pathway was first reported in 1996. In that study, Taxol-induced apoptosis depended on Raf-1 activation (51). Later, an increasing number of studies found that DNA-damaging agents and antitumor compounds induced cell death by activating ERK, which could be rescued using a MEK inhibitor (51–54). In addition, constitutive activation of ERK by death-associated protein kinase 1 or Raf-1 can lead to cell death without other stimuli (55). In this study, by combining bioinformatics analysis and experiments, we demonstrated that downregulation of SYPL1 led to increased apoptosis and ERK activation and vice versa. After cells were treated with selumetinib, a MEK inhibitor, ERK was inactivated, and the number of apoptotic cells was decreased, which demonstrated that knockdown of SYPL1 activated ERK, resulting in cell death.

ROS are a group of chemical reactive molecules with vital roles in cell proliferation and differentiation and include hydroxyl, superoxide, and H_2_O_2_ (56). However, excessive ROS result in oxidative damage to DNA and protein and induce cell death (57). Some studies have reported that ERK activation is involved in this process and that MEK inhibitors suppress ROS-induced cell death (58, 59). Usually, ERK activation is precisely controlled and not prolonged. Sustained activation of ERK is required to induce cell death (60). A review of ERK-induced cell death concluded that ROS-mediated sustained activation of ERK was a crucial mechanism in this process (48). Here, we found that SYPL1-silenced cells had higher levels of ROS and vice versa. Increased ROS contributed to ERK activation and cell apoptosis.

Several antioxidant systems, such as NADPH, SOD, and catalase, assist cells in maintaining redox balance and preventing excessive ROS (61). PPP is a main source of NADPH, which is an ROS scavenger (40). G6PD and PGD are enzymes generating NADPH in the PPP. In addition, there are antioxidant enzymes in peroxisomes, such as SOD1, catalase, PRDX5, and GSTK1 (41). These protect cells from oxidative stress. We analyzed five datasets at the transcription level and found that SYPL1 expression positively correlated with the expression of PPP-related and peroxisome-related antioxidant genes, especially G6PD, PGD, SOD1, and CAT, which was a reflection of the antioxidant status. Generally, SYPL1 expression positively correlated with antioxidant activity, which was consistent with the upregulation of ROS in SYPL1-silenced cells.

We demonstrated that SYPL1 promoted cell proliferation *in vitro* and *in vivo*. Decreased tumor sizes of shSYPL1 cells were observed in a subcutaneous tumor model in BALB/c nude mice. However, in IHC, the expression of SYPL1 did not correlate with the tumor sizes of patients in SYSUCC, which may result from insufficient sample size and sampling error. In addition, PDAC is characterized by desmoplastic stroma, which comprises up to 80% of the tumor mass (62). The subcutaneous xenotransplanted tumor model may not fully reflect the tumor-stroma interaction in the pancreas (63). As a result, the stroma, which comprised the major part of the PDAC in patients, was different in the subcutaneous tumor model in nude mice, which may account for the discrepancy between clinical data and the mouse model. In addition, the effects of SYPL1 on the stroma will be studied.

In summary, this study demonstrated that SYPL1 is upregulated in PDAC, indicating poor prognosis and promoting cell proliferation as well as survival. The mechanism of its anti-apoptotic effect is suppression of ROS-induced ERK activation.

## Data Availability Statement

The raw data supporting the conclusions of this article will be made available by the authors, without undue reservation.

## Ethics Statement

The studies involving human participants were reviewed and approved by the ethics committee of Sun Yat-sen University Cancer Center. The patients/participants provided their written informed consent to participate in this study. The animal study was reviewed and approved by the ethics committee of Sun Yat-sen University Cancer Center. Written informed consent was obtained from the individual(s) for the publication of any potentially identifiable images or data included in this article.

## Author Contributions

SL and YS: study concept and design. YS, XS, and FD: acquisition, analysis, or interpretation of data. YS and XS: drafting of the manuscript. YS, JiW, and JuW: critical revision of the manuscript for important intellectual content. YS, CH, and RW: statistical analysis. SL: obtained funding. XH, YM, FX, KX, and SS: administrative, technical, or material support. SL and JuW: study supervision. All authors have read and approved the final version of the manuscript.

## Conflict of Interest

The authors declare that the research was conducted in the absence of any commercial or financial relationships that could be construed as a potential conflict of interest.

## Abbreviations

SYPL1: synaptophysin-like 1
PDAC: pancreatic ductal adenocarcinoma
ERK: extracellular-regulated protein kinase
ROS: reactive oxygen species
OS: overall survival
HR: hazard ratio
CI: confidence interval
RT-PCR: reverse transcription-quantitative polymerase chain reaction
WB: western blot
KEGG: Kyoto Encyclopedia of Genes and Genomes
SYSUCC: Sun Yat-sen University Cancer Center
IHC: immunohistochemistry.
