# Supplementary material for: SYPL1 Inhibits Apoptosis in Pancreatic Ductal Adenocarcinoma via Suppression of ROS-Induced ERK Activation
Source: Front Oncol. 2020 Sep 15;10:1482. doi: 10.3389/fonc.2020.01482 (PMC7522464; doi:10.3389/fonc.2020.01482)
Supplement: Supplementary file 1 [file Data_Sheet_1.PDF]

Supplementary Figure 1 Pathway analysis (only tumor samples were included). (A) The GSVA score of the cell cycle gene set in SYPL1-high and SYPL1-low samples. (B) The GSVA score of the DNA replication gene set in SYPL1-high and SYPL1-low samples. (C) The GSVA score of the p53 pathway gene set in SYPL1-high and SYPL1-low samples. (D) The GSVA score of the pentose phosphate pathway gene set in SYPL1-high and SYPL1-low samples. (E) The GSVA score of the antioxidant activity gene set in SYPL1-high and SYPL1-low samples. (F) The GSVA score of the peroxisome gene set in SYPL1-high and SYPL1-low samples.

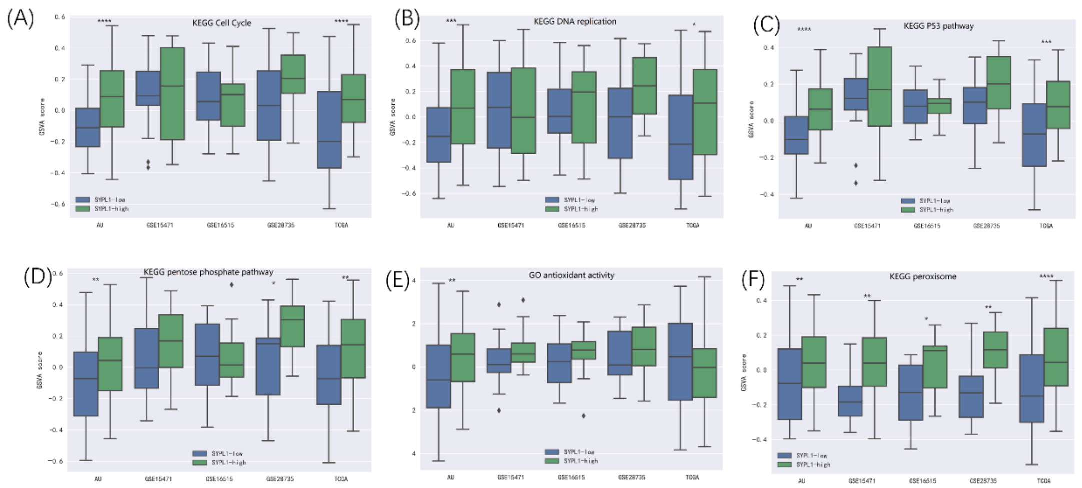

Supplementary Figure 2 Meta-analysis. Pooled results (TCGA, AU, GSE15471, GSE16515 and GSE28735) of (A) p53 pathway activity, (B) cell cycle pathway activity, (C) DNA replication activity and (D) pentose phosphate pathway activity. (E) Pooled results (TCGA, AU and SYSUCC) of prognostic value of SYPL1.

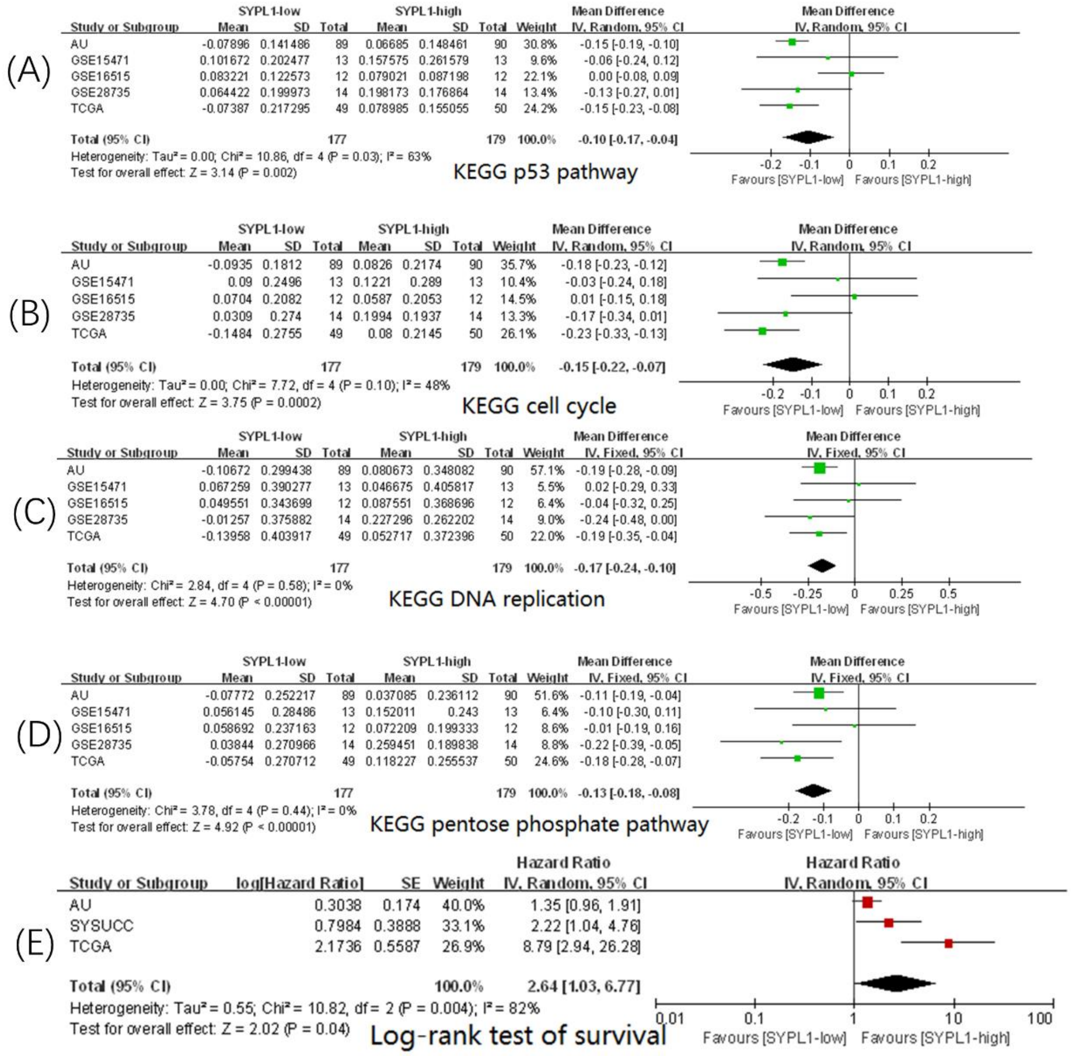

Supplementary Figure 3 (A) Western blotting showed knockdown of SYPL1 using siRNA in BXPC-3 and PANC-1 cells. (B) Colony formation assay showed that knockdown of SYPL1 using siRNA significantly reduced the colony formation ability, while overexpression of SYPL1 rescued the effect of siRNA. (C) CCK8 assay showed that selumetinib did not affect the proliferation of cells.

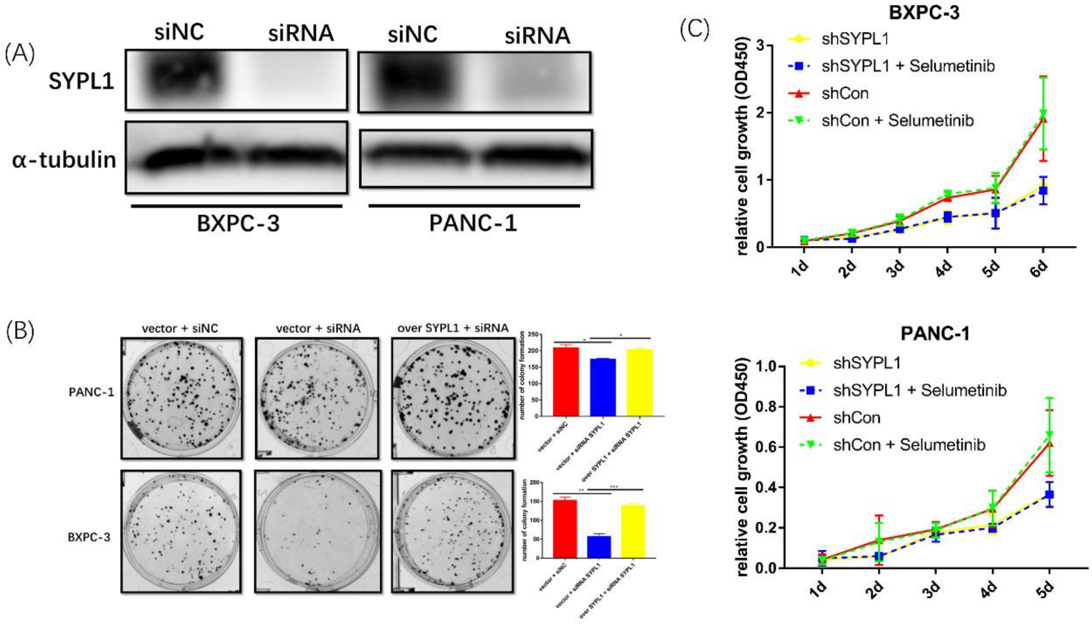

Supplementary Figure 4 (A) repeated western blotting verified that knockdown of SYPL1 activated ERK, and vice versa. (B) Selumetinib suppressed the H_2O_2-induced activation of ERK.

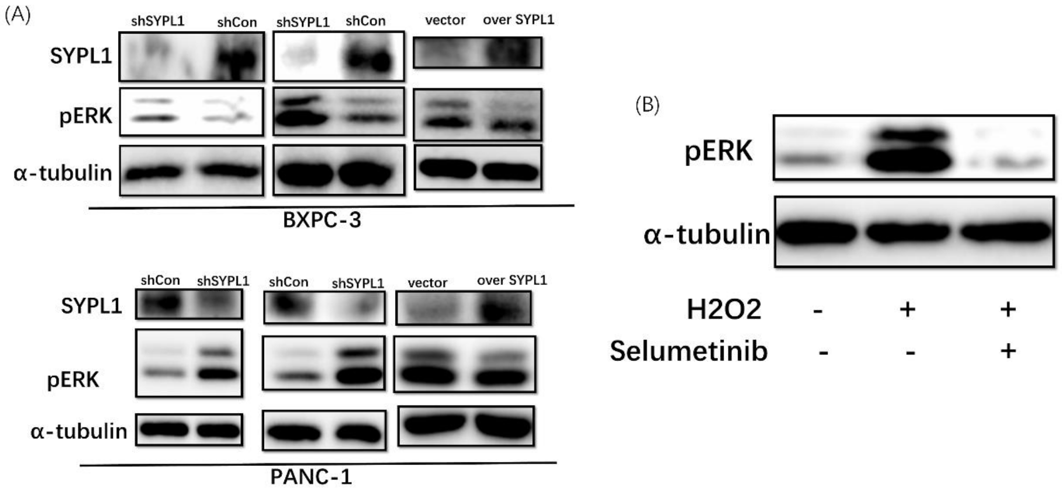

Supplementary Figure 5 The relationship between SYPL1 and tumor size in TCGA.

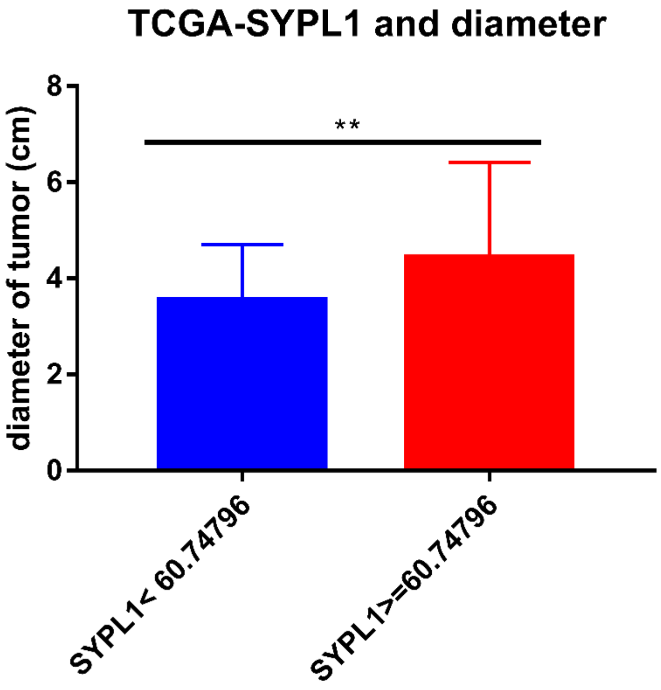

Supplementary Figure 6 Repeated results of flow cytometry showed that overexpression of SYPL1 protected PANC-1 cells from cisplatin-induced apoptosis.

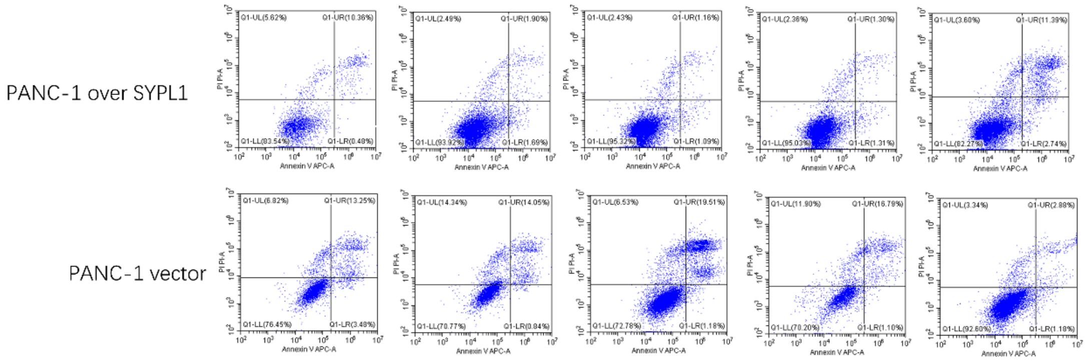

Supplementary Table 1 The number of samples in each dataset.

dataset	tumor	adjacent	normal pancreas
PACA-AU	269	0	0
TCGA	149	0	0
GSE28735	45	45	0
GSE15471	39	39	0
GSE16515	36	16	0
GTEx	0	0	29

Supplementary Table 2 Characteristics of pancreatic ductal adenocarcinoma patients from TCGA.

Clinicopathological features	n	SYPL1 expression low (n=126)	SYPL1 expression high (n=23)	p-values
**Sex**				0.767
male	80	67	13	
female	69	59	10	
**Age**		64.70±10.616	66.96±11.796	0.358
**Tumor site**				0.769
head of pancreas	117	98	19	
body and tail	22	19	3	
unknown	10	9	1	
**Grade**				0.944
G1	21	18	3	
G2	84	71	13	
G3	43	36	7	
G4	1	1	0	
**tumor diamter (cm)**		3.35 (2.80-4.50)	4.00 (3.10-4.85)	0.132
**Nodal status**				0.652
positve	111	93	18	
negative	38	33	5	
**positive lymph node**		2.00 (0-4.00)	2.00 (1.00-3.00)	0.541
